# Human MARF1 is an endoribonuclease that interacts with the DCP1:2 decapping complex and degrades target mRNAs

**DOI:** 10.1093/nar/gky1011

**Published:** 2018-10-26

**Authors:** Tamiko Nishimura, Hana Fakim, Tobias Brandmann, Ji-Young Youn, Anne-Claude Gingras, Martin Jinek, Marc R Fabian

**Affiliations:** 1 Lady Davis Institute for Medical Research, Jewish General Hospital, Montreal, Quebec, Canada; 2 Department of Biochemistry, University of Zurich, Switzerland; 3 Lunenfeld-Tanenbaum Research Institute, Sinai Health System, Toronto, Ontario, Canada; 4 Department of Molecular Genetics, University of Toronto, Toronto, Ontario, Canada; 5 Department of Oncology, McGill University, Montreal, Quebec, Canada

## Abstract

Meiosis arrest female 1 (MARF1) is a cytoplasmic RNA binding protein that is essential for meiotic progression of mouse oocytes, in part by limiting retrotransposon expression. MARF1 is also expressed in somatic cells and tissues; however, its mechanism of action has yet to be investigated. Human MARF1 contains a NYN-like domain, two RRMs and eight LOTUS domains. Here we provide evidence that MARF1 post-transcriptionally silences targeted mRNAs. MARF1 physically interacts with the DCP1:DCP2 mRNA decapping complex but not with deadenylation machineries. Importantly, we provide a 1.7 Å resolution crystal structure of the human MARF1 NYN domain, which we demonstrate is a *bona fide* endoribonuclease, the activity of which is essential for the repression of MARF1-targeted mRNAs. Thus, MARF1 post-transcriptionally represses gene expression by serving as both an endoribonuclease and as a platform that recruits the DCP1:DCP2 decapping complex to targeted mRNAs.

## INTRODUCTION

mRNA degradation is a key process in post-transcriptional regulation of gene expression. One of the major mRNA turnover pathways in eukaryotes initiates with the removal of the mRNA 3′ poly(A) tail by the CCR4-NOT deadenylase complex (1). This is then followed by recruitment of the DCP1:DCP2 decapping complex that hydrolyzes the mRNA 5′-cap structure and commits a transcript for degradation by the 5′-to-3′ exonuclease XRN1 (2). RNA decay proteins localize to processing (P) bodies, discrete cytoplasmic foci that contain the CCR4-NOT complex, as well as decapping proteins including DCP1 and DCP2 (3). The CCR4-NOT deadenylase complex is recruited to targeted mRNAs by a number of gene silencing factors, including the microRNA-induced silencing complex (miRISC) or by RNA-binding proteins, such as TTP (4–9).

While deadenylation most often precedes mRNA decapping, examples do exist of mRNAs that undergo deadenylation-independent degradation. For example, it has been reported to that the yeast ribosomal protein Rps28b recruits the decapping machinery to its own mRNA to bring about decapping in the absence of deadenylation (10). Nonsense mediated decay (NMD) in yeast can also initiate deadenylation-independent decapping followed by mRNA decay (11–13).

**Figure 1.**
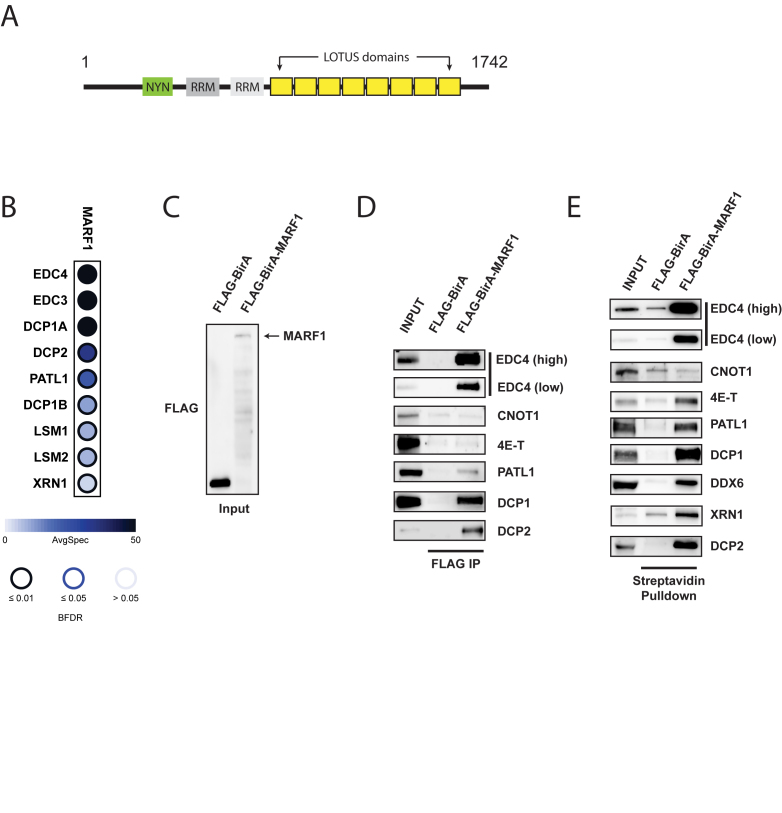
MARF1 interacts with the DCP1:DCP2 mRNA decapping complex. (**A**) Schematic diagram of full-length MARF1. (**B**) Dot plot depicting high-confidence protein interactions identified by affinity purification of FLAG-MARF1 in HEK293 cells. SAINT analysis of two independent experiments was performed and a subset of high-confident preys is presented in this dot plot. Node color represents the average spectral counts, and the node edge color corresponds to the SAINTexpress Bayesian FDR value (BFDR). (**C**) Western blot analysis of lysates derived from HEK293 cells expressing either FLAG-BirA* or FLAG-BirA*-MARF1 and probed with anti-FLAG antibody. (**D**) Immunoprecipitation (IP) of FLAG-BirA* and FLAG-BirA*-MARF1 from benzonase–treated HEK293 cell extracts using anti-FLAG antibody. Immunoprecipitated complexes were separated by SDS-PAGE and probed with antibodies against the indicated proteins. (**E**) Streptavidin pulldowns of biotinylated proteins from benzonase-treated lysates outlined in (C). Precipitated proteins were subjected to SDS-PAGE and probed with antibodies against the indicated endogenous proteins. Inputs represent 2% of total lysates.

Meiosis arrest female 1 (MARF1) is a large protein (1742 aa) that has been shown to be critical for regulating meiotic progression in mouse oocytes (14,15) (Figure 1A). MARF1-null oocytes accumulate Ppp2cb mRNA, the catalytic beta subunit of the major cellular phosphatase PP2A, and specific retrotransposon RNAs, including Long interspersed elements (LINE1) RNA. In addition to its expression in the mammalian germline, MARF1 is also expressed in somatic tissues, including in the developing cerebral cortex where it has been reported to promote neuronal differentiation (16). Notwithstanding the critical role MARF1 plays in mammalian oogenesis, the molecular mechanism underpinning MARF1 function is not understood. Human MARF1 contains two RNA-recognition motif (RRM) domains, and eight minimal LOTUS domains (Figure 1A). It additionally contains a predicted Nedd4BP1 (N4BP1), YacP-like Nuclease (NYN)-like domain. Whether the MARF1 NYN domain exhibits ribonuclease activity has not been investigated.

Here we present data demonstrating that MARF1 engenders deadenylation-independent decay of targeted mRNAs, and provide structural and functional insights into its mechanism of action. Proteomic analysis demonstrates that MARF1 physically interacts with the DCP1:DCP2 mRNA decapping complex but does not associate with the PAN2-PAN3 or CCR4-NOT deadenylase machineries. Interestingly, MARF1 requires its NYN domain, rather than its DCP1:DCP2-interacting motif, in order to degrade a targeted reporter mRNA. We report the crystal structure of the NYN domain, which adopts a PIN (PilT N-terminus) domain-like fold. Furthermore, we show that the NYN domain has intrinsic endoribonuclease activity that can degrade single-stranded RNA *in vitro*, and mutating its catalytic pocket ablates its nuclease activity *in vitro* and silencing through MARF1 *in vivo*. Taken together, our data provide compelling evidence that human MARF1 is a potent effector of mRNA silencing in human cells.

## MATERIALS AND METHODS

### Antibodies

Antibodies against XRN1, PATL1, DCP1, DCP2, EDC4 and DDX6 were from Bethyl Laboratories. CNOT1 antibody was from Proteintech. 4E-T antibody was from Abcam. β-actin antibody was from Cell Signaling. FLAG, HA and MYC antibodies were from Sigma, Covance and Bioshop, respectively.

### Cells and transfections

HeLa cells and Flp-In T-REx HEK293 cells were grown in Dulbecco's modified Eagle's medium (DMEM) supplemented with 10% fetal calf serum, 50 U/ml penicillin, and 50 μg/ml streptomycin. Flp-In T-REx cells were maintained in 100 μg/ml hygromycin B. All plasmid transfections were carried out using polyethylenimine (Polysciences).

### Plasmids

FLAG-BirA*-MARF1 was generated by Gateway cloning of pDONR-MARF1 into pcDEST-pcDNA5-BirA*-FLAG-N-term. Full-length MARF1 and MARF1 fragments were cloned into EcoRI and NotI sites in pCI-**λ**NHA plasmid for tethering experiments. pCI-**λ**NHA-LacZ and reporter plasmids (RL-5BoxB, RL-5BoxB-A114-N40-HhR and FL) are described previously (17,18). MARF1 fragments were cloned into BamHI and SalI sites of pBABE-3XFLAG-puro. MARF1 NYN-mutant and NYN-deletion were generated by site-directed mutagenesis using Phusion Hot-Start II polymerase.

### Affinity purification of FLAG-tagged MARF1 and preparation for mass spectrometry

Cell lines expressing N-terminally FLAG tagged MARF1 constructs were grown to ∼70% confluency in a 15 cm plate, and the bait expression was induced for 24 h with 2 μg/ml doxycycline. Cells were washed cold in PBS and harvested, then snap-frozen. The FLAG AP-MS protocol was adapted from (19) with modifications. Frozen cell pellets were lysed at a 1:4 (pellet weight in g:lysis buffer volume in ml) ratio in ice-cold lysis buffer. Lysis buffer contains 50 mM HEPES–NaOH pH 8.0, 100 mM KCl, 2 mM EDTA, 0.1% NP40 and 10% glycerol. Protease inhibitors (1:500 Sigma protease inhibitor cocktail P8340) were added immediately before use. Frozen cell pellets were thawed in lysis buffer on ice, resuspended by gentle pipetting, and subjected to one cycle of freeze-thaw (dry ice 5 min/ 37°C water bath) to aid in lysis. The samples were sonicated at 4°C using three 10 sec bursts with 3 s pauses at 30% amplitude. 250 units of benzonase nuclease were added, and the lysates were incubated at 4°C for 20 min with rotation. The lysates were then centrifuged at 20 817 g for 20 min at 4°C and the supernatant transferred to tubes containing 25 μl of 50% magnetic anti-FLAG M2 beads slurry (Sigma, M8823) pre-washed in lysis buffer. FLAG immunoprecipitation was allowed to proceed at 4°C for 2 h and 45 min with rotation. Beads were pelleted by centrifugation (1000 rpm for 1 min) and magnetized, and the unbound lysate was aspirated and kept for analysis. The beads were demagnetized and washed with 1 ml lysis buffer and magnetized to aspirate off the wash buffer. The beads were then washed with 1 ml of 20 mM Tris–HCl (pH 8.0) containing 2 mM CaCl_2_, and any excess wash buffer was removed by magnetizing and pipetting off the liquid. The now-dry magnetic beads were removed from the magnet and resuspended in 10 μl of 20 mM Tris–HCl (pH 8.0) containing 1 μg of trypsin (Sigma, T6567) and the mixture was incubated at 37°C with agitation overnight. After the initial incubation, the beads were magnetized and the supernatant was transferred to a fresh tube. An additional 500 ng of trypsin was added in 2.5 μl of 20 mM Tris–HCl (pH 8.0) and the samples were further incubated, without agitation, for 4 h. The sample was acidified with formic acid to a final concentration of 2% and the tryptic digests were stored at −80°C until ready for mass spectrometry analysis.

### Mass spectrometry acquisition using TripleTOF mass spectrometers

A fraction of the digested peptides (5 out of 16 μl) was directly loaded at 400 nl/min onto a 15 cm 100 μm ID emitter tip packed in-house with 3.5 μm Reprosil C18 (Dr Maische). The peptides were eluted from the column at 400 nl/min over a 90 min gradient generated by a 425 NanoLC (Eksigent, Redwood, CA, USA) and analyzed on a TripleTOF™ 6600 instrument (AB SCIEX, Concord, Ontario, Canada). The gradient started at 2% acetonitrile with 0.1% formic acid and increased to 35% acetonitrile over 90 min followed by 15 min at 80% acetonitrile and 15 min at 2% acetonitrile, for a total of 120 min. To minimize carryover between each sample, the analytical column was flushed for 1 h with an alternating sawtooth gradient from 35% acetonitrile to 80% acetonitrile, holding each gradient concentration for 5 min at a flow rate of 1500 nl/min. Analytical column and instrument performance were verified after each sample by analyzing 30 fmol BSA tryptic peptide digest with 60 fmol α-Casein tryptic digest with a short 30 min gradient. MS mass calibration was performed on BSA reference ions between each sample.

MARF1 AP-MS sample was analyzed in Data Dependent Acquisition (DDA) mode by performing one 250 ms MS1 TOF survey scan from 400–1250 Da followed by 20× 100 ms MS2 candidate ion scans from 100 to 2000 Da in high sensitivity mode. Only ions with a charge of 2+ to 4+ that exceeded a threshold of 200 cps were selected for MS2, and former precursors were excluded for 10 s after one occurrence.

### MS data analysis

Mass spectrometry data generated were stored, searched and analyzed using the ProHits laboratory information management system (20). Raw WIFF files were converted to a MGF format using WIFF2MGF converter and to an mxML format using ProteoWizard (21). The searched database contained the human (22) and adenovirus complements of the RefSeq protein database (version 57) supplemented with ‘common contaminants’ from the Max Planck Institute (http://141.61.102.106:8080/share.cgi?ssid=0f2gfuB) and the Global Proteome Machine (GPM; http://www.thegpm.org/crap/index.html) as well as sequences from common fusion proteins and epitope tags. The sequence database consisted of forward and reversed sequences; in total 72 226 sequences were searched. The search engines were Mascot and Comet, with trypsin specificity and two missed cleavage sites allowed. Methionine oxidation and asparagine/glutamine deamidation were set as variable modifications. The fragment mass tolerance was 0.15 Da and the mass window for the precursor was ±35 ppm. The resulting Comet and Mascot search results were individually processed by PeptideProphet (23), and peptides were assembled into proteins using parsimony rules first described in ProteinProphet (24) into a final iProphet (25) protein output using the Trans-Proteomic Pipeline (TPP; Linux version, v0.0 Development trunk rev 0, Build 201303061711). General TPP options were -p0.05 -x20 -PPM - d‘DECOY’, iProphet options were pPRIME and PeptideProphet options were pPAEd. All proteins with a minimal iProphet protein probability of 0.05 were parsed to the relational module of ProHits. Note that for analysis with SAINT, only proteins with iProphet protein probability ≥0.95 were considered. This corresponds to an estimated protein level FDR of ∼0.5%. A minimum of two unique peptide ions was also enforced.

### Interaction proteomics scoring using Significance of Analysis of INTeractome (SAINT)

SAINT calculates, for each prey protein identified in a purification, the probability of true interaction by using spectral counting (semi-supervised modelling, using negative control runs). SAINTexpress (26) analysis was performed using version exp3.3 with two biological replicates of MARF1-FLAG analyzed alongside six negative control runs (compressed to four as previously described; 27), consisting of purifications from cells expressing 3xFLAG-GFP, BirA*-FLAG-GFP or 3xFLAG. All non-human protein contaminants were removed from the SAINT file.

The proteomics dataset consisting of raw files and associated peak list and results files was deposited as a complete submission in ProteomeXchange (identifier PXD007554) through partner MassIVE (MSV000081483). This can be accessed through ftp://massive.ucsd.edu/MSV000081483.

### BioID and co-immunoprecipitation experiments

After an over-night incubation in 50 μM biotin, cell pellets were either lysed on ice for 1 hour in 500 μl of RIPA buffer containing 50 mM Tris–HCl pH 7.5, 150 mM NaCl, 1% NP-40, 1 mM EDTA, 1 mM EGTA, 0.1% SDS, 0.5% sodium deoxycholate, to which a protease inhibitor cocktail (aprotinin, leupeptin and pepstatin) and benzonase (50U) were added immediately before use, or cell pellets were lysed in 500 μl of RIPA buffer at room temperature. Lysates were sonicated and incubated with streptavidin-coupled Agarose (Millipore) for 3 h at 4°C to capture biotinylated proteins. The beads were then pelleted and if the samples were initially lysed in RIPA, the beads were washed with 3× 1 ml with RIPA buffer (not containing protease inhibitors, sodium deoxycholate and benzonase) and 2× 1 ml lysis buffer containing 50 mM HEPES–KOH pH 7.5, 0.1 M KCl, 10% glycerol, 2 mM EDTA and 0.1% NP-40. If the samples were initially lysed in 1× Laemmli, the beads were washed 5× 1 ml RIPA buffer containing 1% Triton X-100, 50 mM Tris–HCl pH 7.5, 150 mM NaCl, 1 mM EDTA, 1 mM EGTA, 0.1% SDS, to which PMSF (1 mM) and sodium deoxycholate (0.5%) were added immediately before use. After the final washes, 40 μl of 2X Laemmli buffer were added to the samples, which were then boiled at 100°C for 8 min to elute.

### Luciferase and Northern blot analysis of reporter mRNA

HeLa cells were lysed 24 h post-transfection in Passive Lysis Buffer (Promega) and RL and FL activities was measured using Dual-Luciferase Assay (Promega). For Northern blot analysis, total RNA was purified on column (BioBasic), resolved on 1.2% denaturing-agarose gels and transferred to nitrocellulose membranes. Probes against RL and FL were generated by PCR, radiolabeled (RadPrime DNA Labeling System; Thermoscientific) and hybridized. Northern blots were visualized and quantified by phosphorimaging (Storm, GE Life Sciences). Representative blots of at least three replicates are shown.

### Recombinant protein purification

The human MARF1 NYN domain, spanning residues 352–500, was expressed as a fusion protein containing an N-terminal Glutathione-S-Transferase (GST) affinity tag followed by the human rhinovirus 3C protease cleavage site and a C-terminal hexahistidine (His_6_) affinity tag. For structure determination by X-ray crystallography, two methionine substitutions (I391M/L457M) were introduced into the DNA sequence of the expression plasmid by site-directed mutagenesis and verified by Sanger sequencing. The protein was expressed in *Escherichia coli* BL21 (DE3) Rosetta 2 (Novagen) cells at 18°C for 16 h after induction. Cells were lysed by sonication in lysis buffer (20 mM Tris pH 8.0, 250 mM KCl) supplemented with 1 mM DTT and the clarified lysate was applied to a 5 ml Glutathione Sepharose 4B cartridge (GE Healthcare). Bound protein was eluted using lysis buffer supplemented with 10 mM reduced glutathione and subsequently dialyzed against lysis buffer in the presence of 3C protease overnight at 4°C to cleave the GST affinity tag. The GST tag was removed by reapplying the cleaved protein onto a 5 ml Glutathione Sepharose 4B cartridge. The protein was further purified by size exclusion chromatography using a Superdex 75 16/600 column (GE Healthcare) in 20 mM Tris pH 7.5, 150 mM KCl, 1 mM DTT.

For selenomethionine (SeMet) labeling, the expression of the MARF1 NYN I391M/L457M mutant protein was carried out in M9 minimal medium supplemented with 1 μg ml^−1^ biotin and 1 μg ml^−1^ thiamine. Bacteria were grown to OD_600_ of 0.8 followed by the addition of an amino acid mix (0.1 mg ml^−1^ Lys, Thr, Phe and 0.05 mg ml^−1^ Leu, Ile, Val, SeMet) allowing the incorporation of SeMet. After 30 min, the temperature was reduced to 18°C and expression was induced by addition of 200 μM isopropyl-β-D-thiogalactopyranoside (IPTG). Purification of SeMet labeled protein was performed as described above.

For in vitro RNA decay assays, wild-type or mutant (containing tandem alanine substitutions for Asp426 and Asp427) MARF1 NYN domains, spanning residues 238–510, were expressed as fusion proteins containing an N-terminal GST affinity tag followed by the human rhinovirus 3C protease cleavage site. Proteins were expressed in *E. coli* BL21 (DE3) Rosetta 2 (Novagen) cells at 37°C for 4 h after induction, purified by glutathione sepharose, and cleaved with HRV 3C Protease (Thermo Scientific) to remove their GST tags.

### Crystallization and Structure determination

The structure of the MARF1 NYN domain was determined by a single-wavelength anomalous diffraction (SAD) experiment, exploiting the anomalous diffraction properties of selenomethionine (SeMet)-substituted MARF1 NYN domain I391M/L457M mutant. Following purification, the protein was concentrated to 17 mg ml^−1^ and crystallized at 20°C using the hanging drop vapor diffusion method by mixing equal volumes (1+1 μl) of protein with a reservoir solution containing 12% (w/v) PEG3350, 0.2 M K_3_-Citrate and 0.1 M Bis-Tris propane, pH 6.5. For cryoprotection, crystals were transferred into 12% (w/v) PEG3350, 0.2 M K_3_-Citrate, 0.1 M Bis-Tris propane, pH 6.5, 25% ethylene glycol and flash-cooled in liquid nitrogen. X-ray diffraction data were collected at beam line X06DA (PXIII) of the Swiss Light Source (Paul Scherrer Institute, Villigen, Switzerland). The crystals diffracted to a resolution of 1.7 Å, belonged to space group *P*4_3_2_1_2 and contained two copies of the protein per asymmetric unit. Diffraction data were processed using XDS (28). Localization of selenium sites, phasing, density modification, and automated building of a preliminary model was carried out using the AutoSol routine implemented in Phenix (29). The initial atomic model was completed by manual building using COOT (30) and refined using Phenix.Refine (31).

### *In vitro* ribonuclease assay

(U)_30_ oligonucleotide (IDT) was radiolabelled with a 5′ phosphate using [γ-^32^P] ATP and PNK enzyme (ThermoFisher). 3′ end-labeled oligonucleotide (U)_30_ was prepared using 5-[^32^P]-pCp and T4 RNA ligase (NEB). Labelled oligonucleotides were each separated from free nucleotides on mini oligo Quick spin columns (Roche) and subsequently gel purified. For nuclease assays, 1 μM of WT or mutant recombinant NYN (238–510) was incubated with 10 pmol of cold (U)_30_ RNA, 0.1 pmol of radiolabelled (5′ or 3′) (U)_30_ in 10 μl reaction volumes containing 20 mM HEPES pH 7.5, 150 mM NaCl, 10% glycerol and 1 mM DTT. Reactions were also supplemented with 3 mM MnCl_2_ or 3 mM MgCl_2_ as described in the figure legends. Reactions were carried out at room temperature for up to 60 min. RNA at each time point was ethanol precipitated and resuspended in 10 μl loading buffer II (ThermoFisher). Samples were boiled at 95°C for 10 min and resolved by electrophoresis on a 15% polyacrylamide and 8 M urea gel and visualized by phosphorimaging (Storm, GE Life Sciences). RNA ladders were prepared by partial alkaline hydrolysis of 5′ end-radiolabeled oligonucleotide (U)_30_ RNA. This was performed in a 5 μl reaction mixture containing 0.1 pmol RNA and 66.7 mM (NaHCO_3_/Na_2_CO_3_, pH 9.5) incubated at 95°C for 5 min. Reactions were terminated by adding 10 μl of loading buffer II (ThermoFisher).

## RESULTS

### MARF1 physically interacts with the DCP1:DCP2 decapping complex

MARF1 was recently reported to localize to processing (P) bodies in somatic cells (32) (33). Thus, we set out to identify MARF1-interacting proteins to determine if MARF1 interacts with any mRNA silencing machineries, including the CCR4-NOT deadenylase complex and/or factors involved in mRNA decapping and decay that localize to P-bodies. To this end, FLAG-tagged MARF1 protein was inducibly expressed from stably-transfected HEK293 cells and affinity-purified from benzonase-treated cell lysates; MARF1-bound proteins were subsequently analyzed by tandem mass spectrometry (MS/MS). Two independent MARF1 replicates were analyzed, alongside negative controls. Using three different groups of negative controls, we performed SAINT (Significance Analysis of Interactome) analysis to obtain a list of high-confidence interactions (Figure 1B and Supplementary Table S1) (34). Among the high-confidence interactors, we found that MARF1 co-precipitates with a number of mRNA decapping factors that localize to P-bodies, including enhancers of mRNA decapping (EDC3 and EDC4), as well as DCP1 and DCP2. We also identified PATL1, components of the LSM1-7 complex and the 5′-3′ exonuclease XRN1. In contrast, we did not detect any subunits of the CCR4-NOT or PAN2-PAN3 deadenylase complexes.

We subsequently performed co-immunoprecipitation from HEK293 cells expressing either a FLAG-tagged negative control or FLAG-tagged MARF1 proteins to validate these interactions (Figure 1C through E; note that the constructs also expresses the biotin ligase BirA*). Full-length FLAG-BirA*-MARF or FLAG-BirA* were affinity-purified through the FLAG epitope under mild lysis and wash conditions, and co-immunoprecipitating proteins were eluted from the beads and resolved by SDS-PAGE followed by western blot analysis (Figure 1D). As in the mass spectrometry experiment, EDC4, PATL1, DCP1A and DCP2 co-precipitated with MARF1, but CNOT1, the central subunit of the CCR4-NOT deadenylase complex, did not. EDC4 has been reported to act as a scaffold upon which DCP1 activates DCP2 for optimal decapping catalytic activity (35), and was previously reported to associate with MARF1 (32).

We further validated these results using BioID labeling coupled to immunoblotting. BioID utilizes an abortive biotin ligase mutant (BirA*) to covalently biotinylate proteins in proximity of BirA*-tagged bait protein, allowing identification of proximal interaction partners *in vivo* (PMID: 22412018). For BioID experiments, HEK293 cells expressing either FLAG-BirA* (control) or FLAG-BirA*-MARF1 proteins were incubated with biotin for 24 hr and lysed under harsh conditions to disrupt protein-protein interactions. Biotinylated proteins were subsequently affinity-purified from benzonase-treated lysates with streptavidin agarose, eluted and analyzed by western blot using antibodies against a number of P-body proteins (Figure 1E). Consistent with our MS data, FLAG-BirA*-MARF1 biotinylated several mRNA decapping and decay factors, including EDC4, PATL1, DCP1, DCP2 and XRN1. In contrast, FLAG-BirA*-MARF1 did not lead to detectable biotinylation of CNOT1. Taken together, these data suggest that MARF1 physically interacts with the DCP1:DCP2 mRNA decapping complex, as well as decapping enhancers and the 5′-3′ exonuclease XRN1, but does not readily associate with the deadenylase machineries.

### The DCP1:DCP2 complex interacts with the MARF1 C-terminus

**Figure 2.**
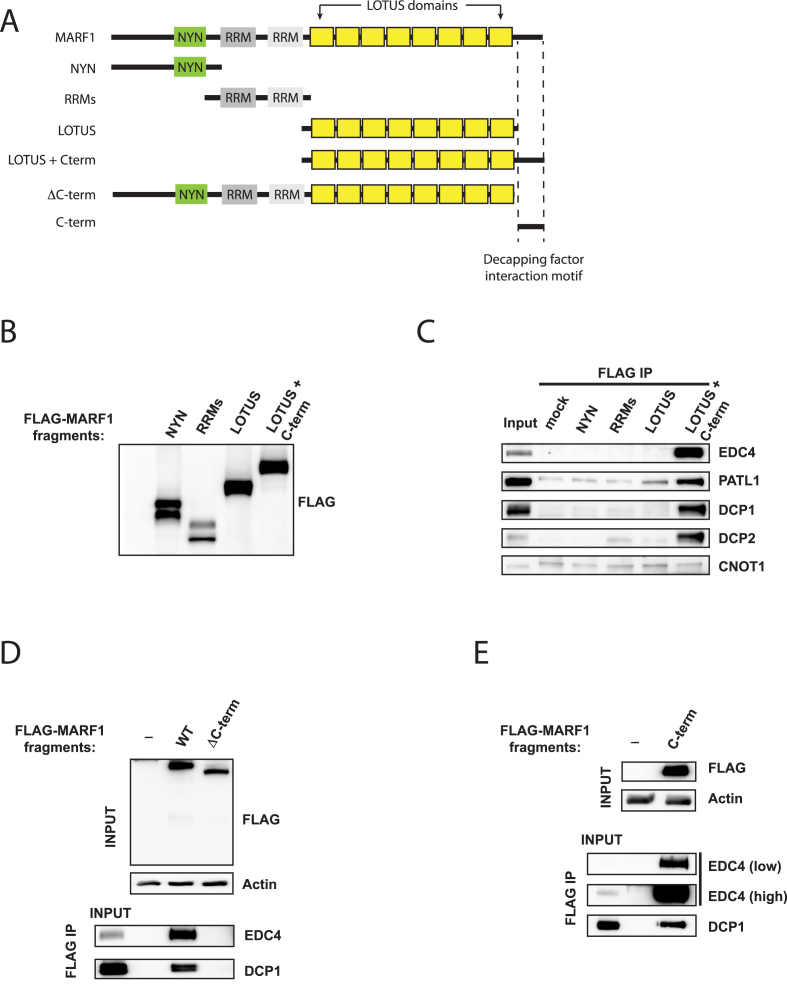
The DCP1:DCP2 complex interacts with the MARF1 C-terminus. (**A**) Schematic diagram of full-length MARF1 and MARF1 fragments used in co-precipitation experiments in B through E. Dashed lines indicated the region required for decapping factor association in (E). (**B**) Western blot analysis of lysates derived from HeLa cells expressing FLAG-tagged MARF1 fragments outlined in (A). (**C**–**E**) Immunoprecipitation (IP) of MARF1 fragments outlined in (A) from benzonase–treated HeLa cell extracts using anti-FLAG antibody. Immunoprecipitated complexes were separated by SDS-PAGE and probed with antibodies against the indicated proteins. Inputs represent 2% of lysates.

Next, we set out to identify which domain of MARF1 interacts with the DCP1:DCP2 decapping complex. To this end, we performed co-immunopreciptiation experiments using a series of FLAG-tagged MARF1 fragments covering the entire MARF1 protein (Figure 2A through E). MARF1 fragments containing the isolated NYN, RRM or LOTUS domains failed to co-precipitate decapping factors (Figure 2C). However, a fragment of MARF1 containing both the LOTUS domains and the C-terminal 172 amino acids of MARF1 (LOTUS + C-term) pulled down EDC4, in agreement with a previous report (32), as well as DCP1A, DCP2 and PATL1. In support of these data, we found that a FLAG-tagged MARF1 protein lacking only the C-terminal region (ΔC-term) did not interact with decapping factors (Figure 2D), whereas a FLAG-tagged C-terminal fragment containing only these 172 residues (C-term) efficiently co-preciptiated both EDC4 and DCP1 (Figure 2E). Taken together, these results indicate that MARF1 contains a C-terminal decapping factor-interaction motif that physically interacts with the DCP1:DCP2 mRNA decapping complex.

### The NYN domain is essential for MARF1-mediated gene silencing

**Figure 3.**
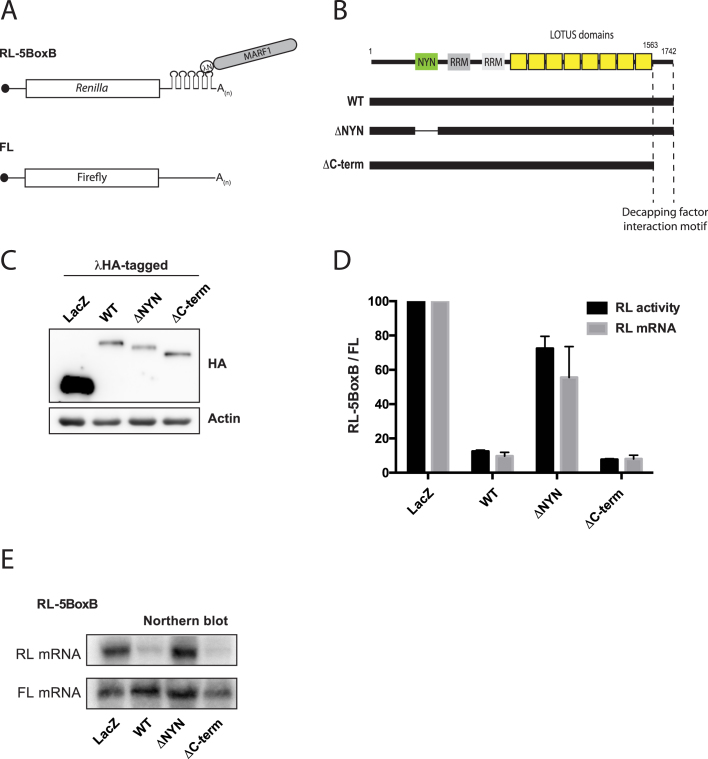
MARF1 NYN domain is required to degrade a target mRNA. (**A**) Schematic diagram of the basic *Renilla* luciferase-encoding mRNA reporter, containing five 19-nt BoxB hairpins, interacting with **λ**N-HA-MARF1. (**B**) Schematic diagram of full-length MARF1 and MARF1 fragments used in tethering assays. (**C**) Expression levels of λNHA-tagged fusion proteins, as determined by western analysis using anti-HA and anti-actin antibodies. (**D**) RL activity detected in extracts from HeLa cells expressing the indicated proteins. Cells were cotransfected with constructs expressing the RL-5BoxB reporter, FL, and indicated fusion proteins. Histograms represented normalized mean values of RL activity and mRNA levels from a minimum of three experiments. RL activity values seen in the presence of λNHA-LacZ were set as 100. (**E**) Representative Northern blot of RL-5BoxB and FL mRNAs levels.

A previous study reported that MARF1 knock-out mouse oocytes display increased RNA expression of several retrotransposons, including the LINE1 RNA (15). As MARF1 physically interacts with the mRNA decapping complex, we next sought to investigate whether MARF1 has the ability to silence a targeted mRNA. To this end, we took advantage of the bacteriophage **λ**N-BoxB tethering system in HeLa cells to determine whether MARF1 can silence a *Renilla* luciferase (RL) reporter mRNA when tethered to its 3′UTR (36) (Figure 3A). **λ**NHA-tagged MARF1 constructs were co-transfected with an RL construct containing 5 BoxB hairpins in its 3′UTR and a Firefly luciferase (FL) construct as a transfection control (Figure 3B through E). Indeed, when full-length **λ**NHA-MARF1 was tethered to the reporter transcript, Renilla luciferase expression was reduced relative to that in cells expressing the **λ**N-peptide fused to LacZ (Figure 3D). This repression appeared to be at the level of mRNA expression or stability, as tethering MARF1 to the reporter mRNA significantly reduced its levels, as shown by Northern blot analysis (Figure 3D and E). In contrast to wild-type MARF1, a MARF1 mutant lacking the NYN domain (MARF1^ΔNYN^) was unable to efficiently silence the reporter mRNA. Moreover, the reporter mRNA remained stable when tethered to MARF1^ΔNYN^. Interestingly, a MARF1 mutant lacking the decapping factor interaction motif (ΔC-term) silenced a reporter RNA as well as wild-type MARF1. Taken together, these data suggest that MARF1 post-transcriptionally represses gene expression by initiating the decay of a targeted mRNA. Moreover, our data suggest that MARF1 requires its NYN domain in order to engender the decay of a targeted mRNA.

### The MARF1 NYN domain is a *bona fide* endoribonuclease

**Figure 4.**
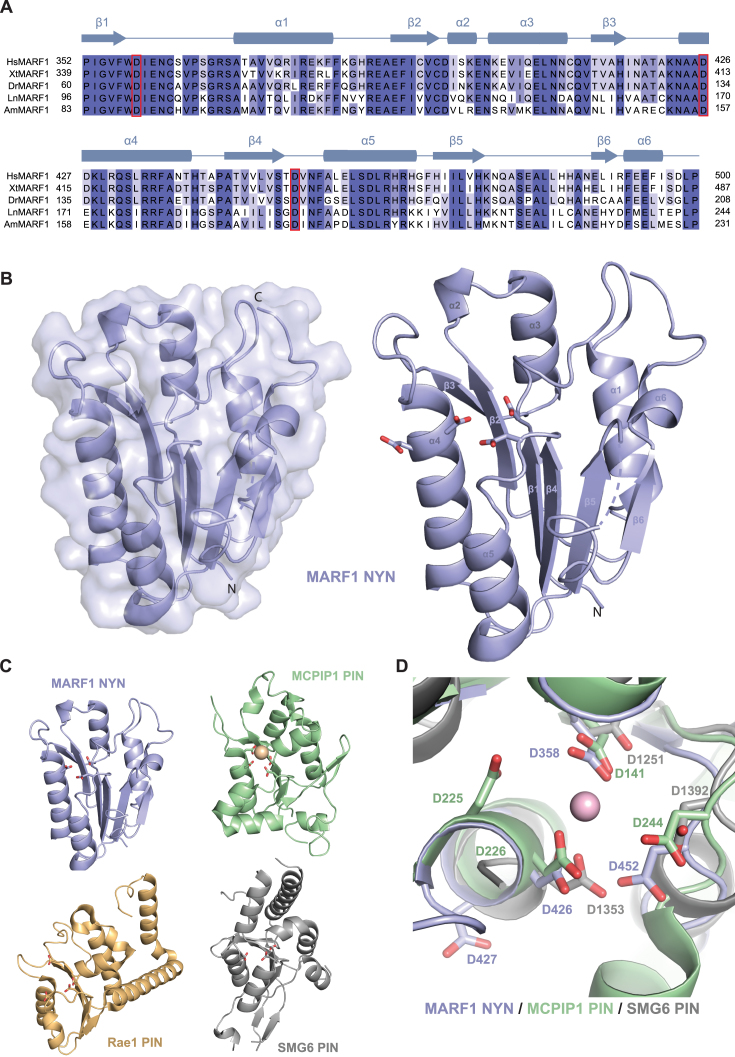
Structure of the MARF1 NYN domain. (**A**) Comparative sequence analysis of the NYN domains of human (Hs), *Xenopus tropicalis* (Xt), *Danio rerio* (Dr), *Lasius niger* (Ha) and *Apis mellifera* (Am) MARF1 orthologs. The multiple sequence alignment was generated using MAFFT version 7 and formatted using Jalview (49,50). Secondary structure elements with corresponding numbering are indicated above the sequence. Invariant residues are coloured dark blue while conservative substitutions are depicted in shades of light blue. (**B**) Left, Crystal structure of the MARF1 NYN domain shown in cartoon and surface representations. Right, cartoon representation of the MARF1 NYN domain, with invariant active site residues depicted in stick format. (**C**) Structural superpositions of the human MARF1 NYN domain with the NYN domain of Bacillus subtilis YacP/Rae1 (PDB 5MQ8), and the PIN domains of human SMG6 (PDB 2HWW) and MCPIP1 (PDB 3V34). The structures were superimposed using the DALI server (51) and are shown in identical orientations. (**D**) Zoom-in view of the NYN domain ribonuclease active site of MARF1, overlaid with the structures of human SMG6 and MCPIP1 PIN domains. Invariant active site residues are shown in stick format. The bound magnesium ion present in the MCPIP1 structure is depicted as a purple sphere.

The MARF1 NYN domain belongs to a superfamily that comprises the PIN, NYN and FLAP/5′-3′ exonuclease domains, and includes the eukaryotic Nedd4-binding proteins and bacterial YacP-like nucleases (14,37). The domain is highly conserved among vertebrates and several insect species (Figure 4A). To gain structural insights into the MARF1 NYN domain, we crystallized a human MARF1 protein construct encompassing amino acid residues 352–500 and determined its structure at a resolution of 1.7 Å by single-wavelength anomalous diffraction (Figure 4B). Although the NYN domain crystallized as a dimer in the asymmetric unit of the crystal, both the wild-type NYN domain and the mutated protein used for crystallization (I391M/L457M) are monomeric in solution, as determined by size exclusion chromatography (Supplementary Figure S1). The MARF1 NYN domain consists of a central six-stranded parallel beta-sheet flanked by pairs of alpha-helices on both sides, in contrast to the previously determined structure of the NYN domain of *Bacillus subtilis* YacP/Rae1, whose central beta-sheet contains only five strand (38). Despite lack of extensive sequence conservation, the MARF1 NYN domain exhibits substantial structural similarity to canonical PIN domains of ribonucleases MCPIP1 (39) and SMG6 (40), superimposing with root-mean-square deviations of 3.0 Å (over 96 Cα atoms) and 3.4 Å (94 Cα atoms), respectively (Figure 4C, D). A prominent feature of the molecular surface of the MARF1 NYN domain is a highly conserved negatively charged patch formed by Asp358, Asp426, Asp427 and Asp452 (Figure 4B, C). Similar clusters of acidic amino acids define the active sites of PIN domain ribonucleases, including SMG6 and MCPIP1, in which they coordinate a divalent metal ion that in turn coordinates the substrate RNA and activates a water molecule for nucleophilic attack on the scissile phosphodiester group (39,40). Importantly, these amino acids are strictly conserved in MARF1 orthologs and map to equivalent residues in the SMG6 and MCPIP1 domain structures (Figure 4D and Supplementary Figure S2). Crucially these residues are not present in the SMG5 PIN domain, which has been shown to lack RNase activity (Supplementary Figure S2) (40). Together, these structural observations strongly suggest that the MARF1 NYN domain is a catalytically competent ribonuclease.

**Figure 5.**
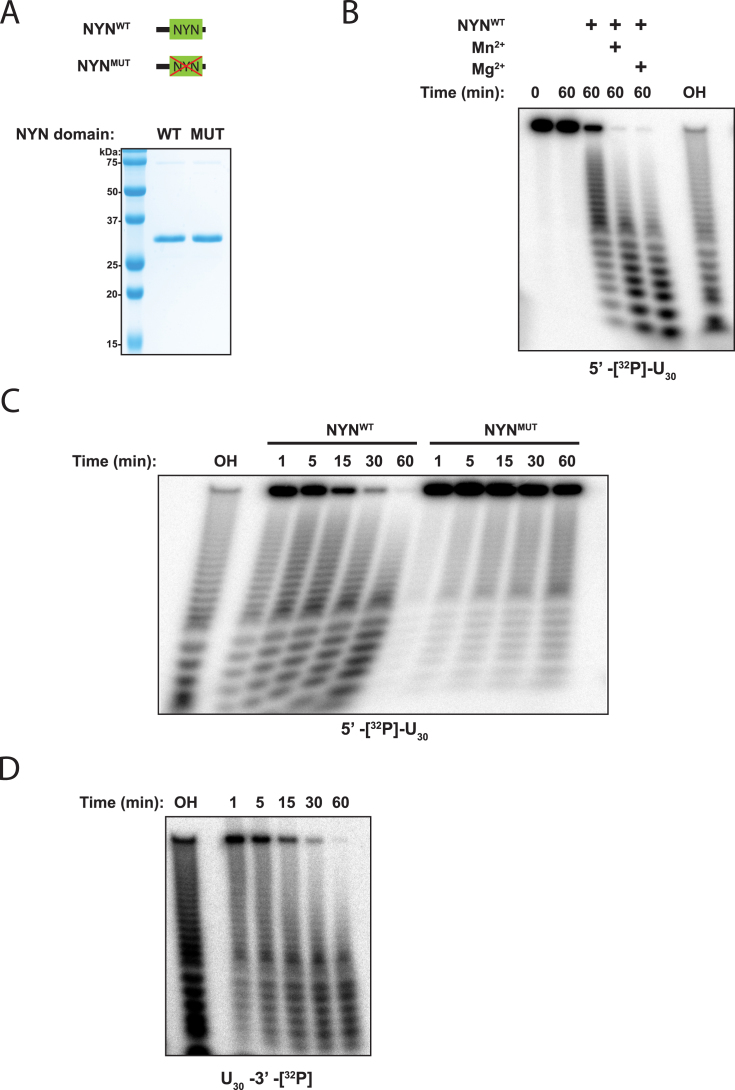
The MARF1 NYN displays endoribonuclease activity *in vitro*. (**A**) Upper panel: Schematic representation of the wild-type and mutant (MUT) MARF1 NYN proteins used for *in vitro* degradation assays. Mutant MARF1 protein contains tandem alanine substitutions for Asp426 and Asp427. Lower panel: Coomassie-stained SDS-PAGE gel of recombinant wild-type (WT) or mutant (MUT) MARF1 NYN proteins. (**B**) NYN^WT^ protein was incubated with a 5′-^32^P-end-labelled U_30_ oligonucleotide in the presence or absence of 3 mM Mn^2+^ or Mg^2+^. RNA was extracted at defined time points and resolved via denaturing PAGE. RNA signals were visualized by autoradiography. OH: hydrolysis ladder. (**C**) Time course analysis of NYN^WT^ and NYN^MUT^ proteins incubated with a 5′-^32^P-end-labelled U_30_ oligonucleotide in the presence of Mn^2+^. OH: hydrolysis ladder. (**D**) NYN^WT^ protein was incubated in the presence of Mn^2+^ with a 3′-^32^P-end-labelled U_30_ oligonucleotide. RNA was extracted at defined time points and resolved via denaturing PAGE. OH: hydrolysis ladder.

Since the MARF1 NYN domain is essential for MARF1 to degrade a reporter RNA *in vivo* (Figure 3D), it was pertinent to investigate whether the MARF1 NYN displays ribonuclease activity. To directly test this, we generated a recombinant MARF1 NYN domain (NYN^WT^) (Figure 5A) and incubated it with a 5′ [^32^P]-end-labeled single stranded (U)_30_ oligonucleotide. The integrity of the RNA was then analyzed by denaturing polyacrylamide-gel electrophoresis (PAGE) followed by autoradiography (Figure 5B). Our crystal structure revealed that MARF1 contains a conserved active site pocket that is likely capable of binding a divalent ion necessary for RNase activity (Figure 4B). In keeping with these data, purified NYN protein is partially active on its own, but efficiently degrades the single-stranded oligo in the presence of manganese or magnesium (Figure 5B). In order to determine whether residues in the active site pocket are important for NYN enzymatic activity, we generated a NYN mutant in which Asp426 and 427 were mutated in tandem to alanines (NYN^MUT^) (Figure 5A). The nuclease activity of NYN^MUT^ was strongly reduced as compared to NYN^WT^ (Figure 5C). As a number of PIN domains act as endonucleases (41), we wished to determine if the MARF1 NYN domain displays endonuclease activity. This this end, we incubated recombinant NYN^WT^ protein with a 3′[^32^P]-end-labeled (U)_30_ (Figure 5D), and observed a similar decay pattern for the 3′ end-labelled oligo as compared to the 5′ end-labelled RNA. Taken together, these data suggest that the MARF1 NYN domain is an endoribonuclease.

### MARF1 NYN domain endonuclease activity promotes mRNA decay in vivo

**Figure 6.**
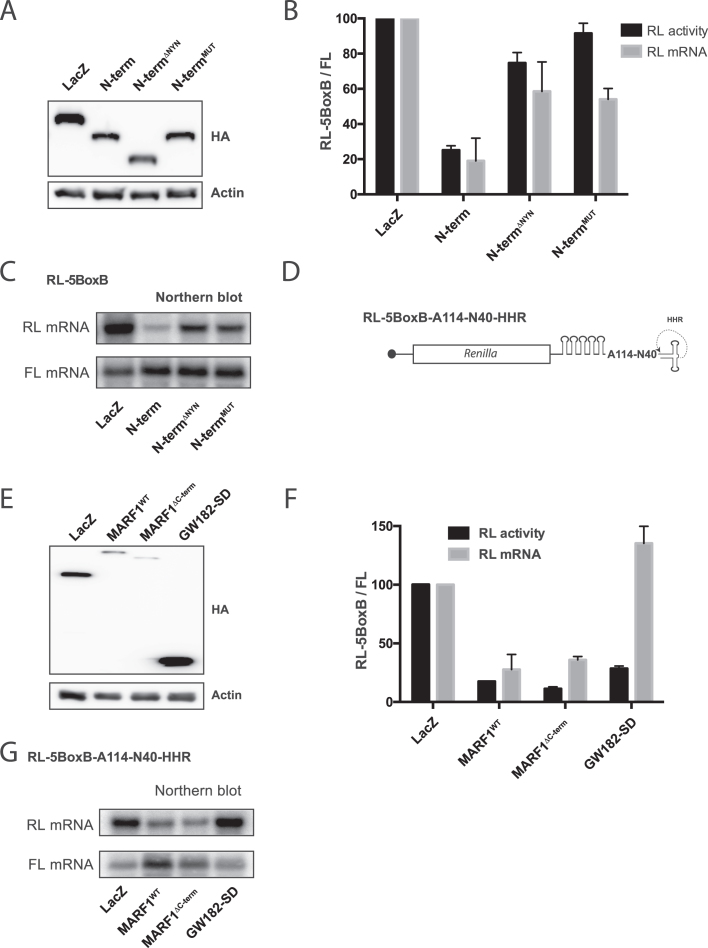
MARF1 NYN displays endonuclease activity *in vivo*. (**A** and **E**) Expression levels of λNHA-tagged fusion proteins, as determined by Western analysis using anti-HA and anti-actin antibodies. (**B**) RL activity detected in extracts from HeLa cells expressing the indicated proteins. Cells were cotransfected with constructs expressing the RL-5BoxB reporter, FL, and indicated fusion proteins. Histograms represented normalized mean values of RL activity and mRNA levels from a minimum of three experiments. RL activity values seen in the presence of λNHA-LacZ were set as 100. (**C**) Representative Northern blot of RL-5BoxB and FL mRNAs levels for (B). (**D**) Schematic diagram of the Renilla luciferase-encoding mRNA reporter, containing five 19-nt BoxB hairpins, a 114 nt internal poly(A), a 40 nt linker and a self-cleaving hammerhead ribozyme (HHR). HHR cleavage site is denoted by an arrow. (**F**) Cells were cotransfected with constructs expressing the RL-5BoxB-A114-N40-HHR reporter, FL, and indicated fusion proteins. Histograms represented normalized mean values of RL activity and mRNA levels from a minimum of three experiments. RL activity values seen in the presence of λNHA-LacZ were set as 100. (**G**) Representative Northern blot of RL-5BoxB-A114-N40-HHR and FL mRNAs levels for (F).

To determine if the activity of the NYN domain is required for MARF1-mediated silencing *in vivo*, we deleted (N-term^ΔNYN^) or mutated (N-term^MUT^) the NYN domain in the context of the MARF1 N-terminal fragment and investigated silencing of the tethered reporter mRNA (Figure 6A–C). Indeed, either deletion of the NYN domain or mutation of aspartates 426 and 427 in the N-terminal MARF1 fragment impaired its ability to silence and degrade a targeted reporter mRNA *in vivo*.

To ascertain whether MARF1 promotes mRNA decay *in vivo* in a deadenylation-dependent or -independent manner, we took advantage of a RL-5BoxB reporter that contains a self-cleaving hammerhead ribozyme (HHR) to generate an internalized 114 nucleotide poly(A) stretch (Figure 6D) (42). Importantly, this reporter mRNA can be translationally silenced by the CCR4-NOT deadenylase complex, but it remains stable because a 3′ stretch of non-A nucleotides (N40) blocks deadenylation and subsequent decay. We tethered **λ**NHA-tagged full-length MARF1 (MARF1^WT^) or a MARF1 deletion mutant lacking the C-terminal decapping factor-interacting motif (MARF1^ΔC-term^) to this reporter and subsequently measured luciferase activity and reporter mRNA abundance (Figure 6E–G). As a negative control, we tethered the GW182 silencing domain (GW182-SD), which interacts with the CCR4-NOT deadenylase complex has been previously shown to translationally repress this reporter mRNA in the absence of deadenylation and decay (43). Tethering MARF1^WT^, MARF1^ΔC-term^ or the GW182-SD to our reporter mRNA resulted in similar reduction in luciferase activity, as compared to LacZ tethering (Figure 6F). In agreement with previous reports, λNHA-GW182-SD did not change the levels of the reporter mRNA (Figure 6F and G). However, tethering MARF1^WT^ or MARF1^ΔC-term^ to this reporter mRNA significantly reduced its levels. These therefore data suggest that the MARF1 NYN domain displays endoribonuclease activity, which is able to engender deadenylation-independent mRNA decay.

## DISCUSSION

Here we present data establishing the MARF1 RNA binding protein an effector of post-transcriptional control and we shed light upon its mechanism of action. Our proteomic, structural and biochemical methods as well as functional assays demonstrate that MARF1 is an endoribonuclease that interacts with the DCP1:DCP2 complex and brings about the decay of targeted mRNAs.

### MARF1 recruits the DCP1:DCP2 mRNA decapping complex

The major pathway for mRNA turnover in eukaryotes initiates with removal of the 3′ poly(A) tail by the PAN2-PAN3 and CCR4-NOT deadenylase complexes (44). This is followed by hydrolysis of the 5′ cap structure by the DCP1:DCP2 decapping complex and 5′-3′ decay of the mRNA body via the XRN1 exonuclease. Multiple RNA binding or RNA-associated proteins promote mRNA decay of targeted transcripts by physically interacting with the CCR4-NOT complex to bring about the deadenylation and subsequent decay of targeted mRNAs. These include GW182, TTP, YTHDF2, DND1 and Nanos (5,7–9,45,46). Our results demonstrate that MARF1 also engenders the decay of a target mRNA. However, in contrast to these RNA binding proteins, our results suggest that MARF1 physically interacts with the DCP1:DCP2 mRNA decapping complex rather than with deadenylase machineries. Mass spectrometry analysis of MARF1-interacting proteins identified the DCP2 decapping enzyme and DCP1, enhancers of mRNA decapping (EDC3 and EDC4), PATL1, components of the LSM1-7 complex (LSM1 and 2) and the 5′-3′ exonuclease XRN1. These results were validated using BioID proximity-labeling coupled to immunoblotting indicating that these interactions exist *in vivo*, rather than manifesting post cell lysis. We next show that the distal C-terminal region of MARF1 acts as the docking site for the decapping machinery (Figure 2). These data are in agreement with a previous study in which ectopically expressed EDC4 was found to associate with overexpressed MARF1 (32). However, our data indicate that a MARF1 mutant that does not interact with the decapping machinery also destabilizes a targeted reporter mRNA (Figures 3 and 6), suggesting that the DCP1:DCP2 complex may be recruited in trans to degrade the 5′ fragment.

### The MARF1 NYN domain displays endoribonuclease activity

MARF1 is critical for generating fertilization-competent oocytes (15). MARF1 knockout mouse oocytes prematurely arrest in meiosis and have been reported to display altered gene expression profiles, including LINE1 retrotransposon accumulation. Here we show that MARF1 post-transcriptionally represses gene expression by degrading targeted mRNAs (Figures 3 and 6), and requires it NYN domain in order to do so. The crystal structure of the NYN domain shows that it adopts a PIN-like fold that is structurally similar to that of other endoribonucleases, including Rae1, MCPIP1/Regnase and SMG6 (Figure 4) (38,40,47). In agreement with the crystal structure, a recombinant MARF1 fragment that includes the NYN domain displays intrinsic endoribonuclease activity *in vitro* (Figure 5).

**Figure 7.**
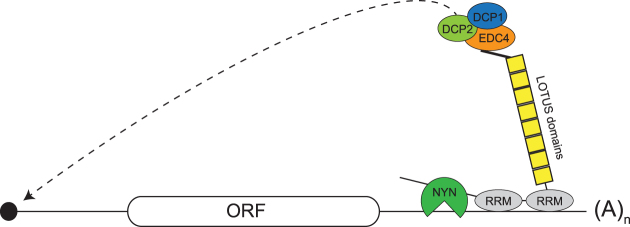
Model of MARF1-mediated mRNA decay. MARF1 binds to a target mRNA via its RRMs. The MARF1 NYN endonuclease cleaves the target mRNA, which is subsequently decapped by the DCP1:DCP2 complex and degraded by XRN1 (not shown). Of note, MARF1-binding sites on targeted mRNAs have yet to be experimentally validated.

In line with our *in vitro* degradation experiments, MARF1 requires its endoribonuclease activity to silence a target mRNA in vivo, as a catalytically inactive MARF1 protein fails to efficiently silence a reporter mRNA in cells (Figure 6A–C). While many mRNAs decay in a deadenylation-dependent manner (i.e. miRNA targeted mRNAs), our data suggests that MARF1 mediates deadenylation-independent mRNA decay (Figure 6D–G). Ultimately, our results support a model whereby MARF1-targeted mRNAs are first cleaved by the NYN domain, and are subsequently decapped and degraded by the DCP1:DCP2 decapping complex and XRN1, respectively (Figure 7).

MARF1 is evolutionarily conserved in vertebrates and selected insects (Figure 4A). A *Drosophila melanogaster* (*Dm*) MARF1-like protein also exists that contains multiple LOTUS domains, but lacks a NYN domain (48). Whether *Dm* MARF1 functions in a similar manner to human MARF1 remains to be established. The LOTUS domains in MARF1 are also found in other germline proteins, including Tudor domain containing 5 (TDRD5), TDRD7 and Oskar (48). A recent study showed that the LOTUS domains of TDRD5, TDRD7 and Oskar bind to and stimulate the germline RNA helicase Vasa (48). In contrast, MARF1 LOTUS domains lack a C-terminal extension found in TDRD5/7 and Oskar, and do not associate with Vasa in flies. Thus, the function of the MARF1 LOTUS domains remains elusive. While the LOTUS domains themselves do not associate with the decapping machinery, they may function to orient the DCP1:DCP2 complex towards the cap structure to facilitate decapping.

In summary, our study identifies MARF1 as an effector of mRNA decay and provides structural and functional insights into its mode of action. We show that MARF1 physically interacts with the DCP1:DCP2 RNA decapping complex and utilizes its PIN-like NYN endonuclease domain to degrade target mRNAs. In addition to its expression in the mammalian germline, MARF1 is also expressed in somatic tissues, including in the developing cortex where its expression has been reported to promote neuronal differentiation (16). Importantly, a MARF1 N-terminal truncation mutant that lacks the NYN domain could not promote neurogenesis. Taken together with our data, this suggests that MARF1-mediated mRNA decay plays an important role in a number of somatic cell populations.

## DATA AVAILABILITY

The coordinates and structure factors for the MARF1 NYN domain structure have been deposited in the Protein Data Bank under accession code 6FDL. The proteomics dataset was deposited as a complete submission in ProteomeXchange (identifier PXD007554) through partner MassIVE (MSV000081483). This can be accessed through ftp://massive.ucsd.edu/MSV000081483.

## Supplementary Material

Supplementary Data
