# Supplementary material for: Human MARF1 is an endoribonuclease that interacts with the DCP1:2 decapping complex and degrades target mRNAs
Source: Nucleic Acids Res. 2018 Oct 26;46(22):12008–21. doi: 10.1093/nar/gky1011 (PMC6294520; doi:10.1093/nar/gky1011)
Supplement: Supplementary Data [file gky1011_supplemental_files.zip › Nishimura et al., 2018 Supplementary Table 2.docx]

**Supplementary Table 2**

|  | MARF1 NYN |
| --- | --- |
| **Dataset** | **SeMet SAD** |
| X-ray source | SLS X06DA (PXIII) |
| Space group | *P*4_3_2_1_2 |
| Cell dimensions |  |
| *a, b, c* (Å) | 96.87, 96.87, 63.10 |
| *α, β, γ* (^o^) | 90.0, 90.0, 90.0 |
| Wavelength (Å) | 0.97940 |
| Resolution (Å)* | 48.43-1.75 (1.81-1.75) |
| *R*_merge_* | 0.108 (1.029) |
| CC1/2* | 0.997 (0.841) |
| *I*/σ*I** | 18.0 (1.7) |
| Observations* | 619812 (47348) |
| Unique reflections* | 30788 (2956) |
| Multiplicity* | 20.1 (15.9) |
| Completeness (%)* | 99.8 (98.3) |
| **Refinement** |  |
| Resolution (Å) | 48.43-1.75 |
| No. reflections | 30770 (2956) |
| *R_work_ / R*_free_ | 0.190/0.219 |
| **No. atoms** |  |
| Protein | 2399 |
| Water | 160 |
| **B-factors** |  |
| mean | 35.01 |
| Protein | 34.98 |
| Water | 35.49 |
| **R.m.s. deviations** |  |
| Bond lengths (Å) | 0.007 |
| Bond angles (^o^) | 0.890 |
| **Ramachandran plot** |  |
| % favored | 99.3 |
| % allowed | 0.7 |
| % outliers | 0.0 |
