# Supplementary material for: Human MARF1 is an endoribonuclease that interacts with the DCP1:2 decapping complex and degrades target mRNAs
Source: Nucleic Acids Res. 2018 Oct 26;46(22):12008–21. doi: 10.1093/nar/gky1011 (PMC6294520; doi:10.1093/nar/gky1011)
Supplement: Supplementary Data [file gky1011_supplemental_files.zip › Supplementary Material.pdf]

Supplementary Figure 1. MARF1 NYN domain is monomeric in solution.

Size exclusion chromatograms of wild-type MARF1 NYN domain (left) and the I391M/L457M mutant used for crystal structure determination (right). Both proteins were analyzed using a Superdex 75 26/600 column (GE Healthcare) and eluted in 20 mM Tris pH 7.5, 150 mM KCl, 1 mM DTT.

Supplementary Figure 2. MARF1 NYN domain is structurally similar to MCPIP1 and SMG6 PIN domains (A) Structure-based sequence alignment of the NYN domains of human MARF1 and Bacillus subtilis YacP/Rae1 (PDB 5MQ8) and the PIN domains of human MCPIP1 (PDB 3V34) and SMG6 (PDB 2HWW). The alignment was generated by submitting the respective PDB coordinates to the DALI server (49). Secondary structure elements of MARF1 NYN with corresponding numbering are indicated above the sequence. Invariant residues are coloured dark blue while conservative substitutions are depicted in shades of light blue. Asp residues in equivalent positions composing the active sites are highlighted by red boxes. (B-C) Structural superpositions of the human MARF1 NYN domain with the PIN domain of human SMG6 (PDB 2HWW) and MCPIP1 (PDB 3V34) respectively. The structures were superimposed using the DALI server (49) and are shown in identical orientations. Invariant active site residues are shown in stick format. The bound magnesium ion present in the MCPIP1 structure is depicted as a purple sphere.

Supplementary Table 1

	MARF1 NYN
**Dataset**	**SeMet SAD**
X-ray source	SLS X06DA (PXIII)
Space group	$P4_32_12$
Cell dimensions	
a, b, c (Å)	96.87, 96.87, 63.10
α, β, γ (°)	90.0, 90.0, 90.0
Wavelength (Å)	0.97940
Resolution (Å)*	48.43-1.75 (1.81-1.75)
R_{merge}*	0.108 (1.029)
CC1/2*	0.997 (0.841)
$I/\sigma I$*	18.0 (1.7)
Observations*	619812 (47348)
Unique reflections*	30788 (2956)
Multiplicity*	20.1 (15.9)
Completeness (%)*	99.8 (98.3)
**Refinement**	
Resolution (Å)	48.43-1.75
No. reflections	30770 (2956)
R_{work} / R_{free}	0.190/0.219
**No. atoms**	
Protein	2399
Water	160
**B-factors**	
mean	35.01
Protein	34.98
Water	35.49
**R.m.s. deviations**	
Bond lengths (Å)	0.007
Bond angles (°)	0.890
**Ramachandran plot**	
% favored	99.3
% allowed	0.7
% outliers	0.0
