# Supplementary figures and images for: Human MARF1 is an endoribonuclease that interacts with the DCP1:2 decapping complex and degrades target mRNAs
Source: Nucleic Acids Res. 2018 Oct 26;46(22):12008–21. doi: 10.1093/nar/gky1011 (PMC6294520; doi:10.1093/nar/gky1011)

## MARF1-NYN WT

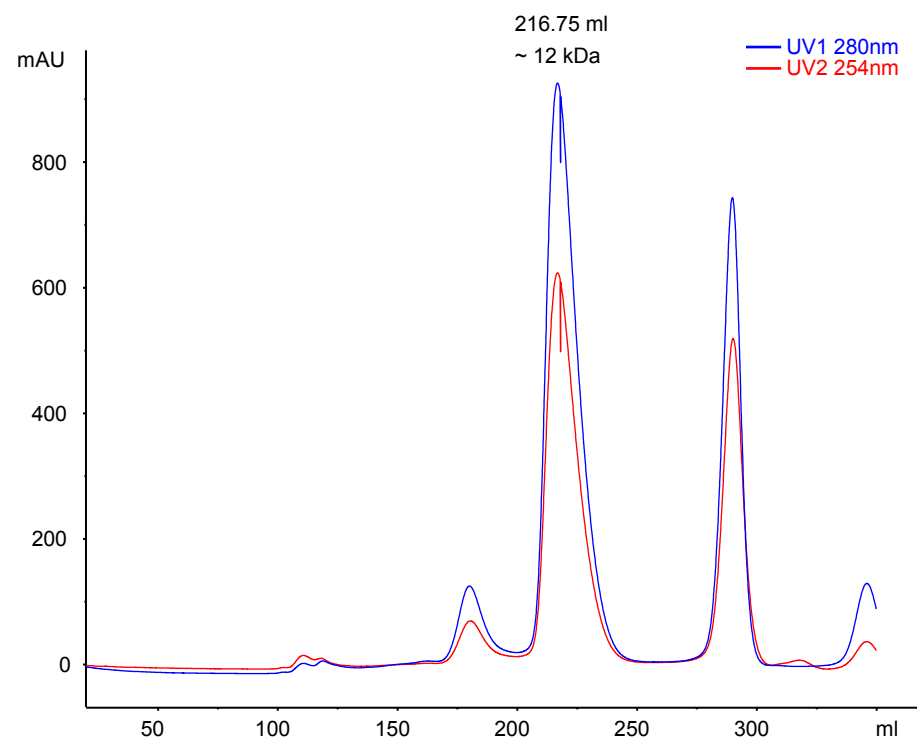

## MARF1-NYN (I391M / L457M)

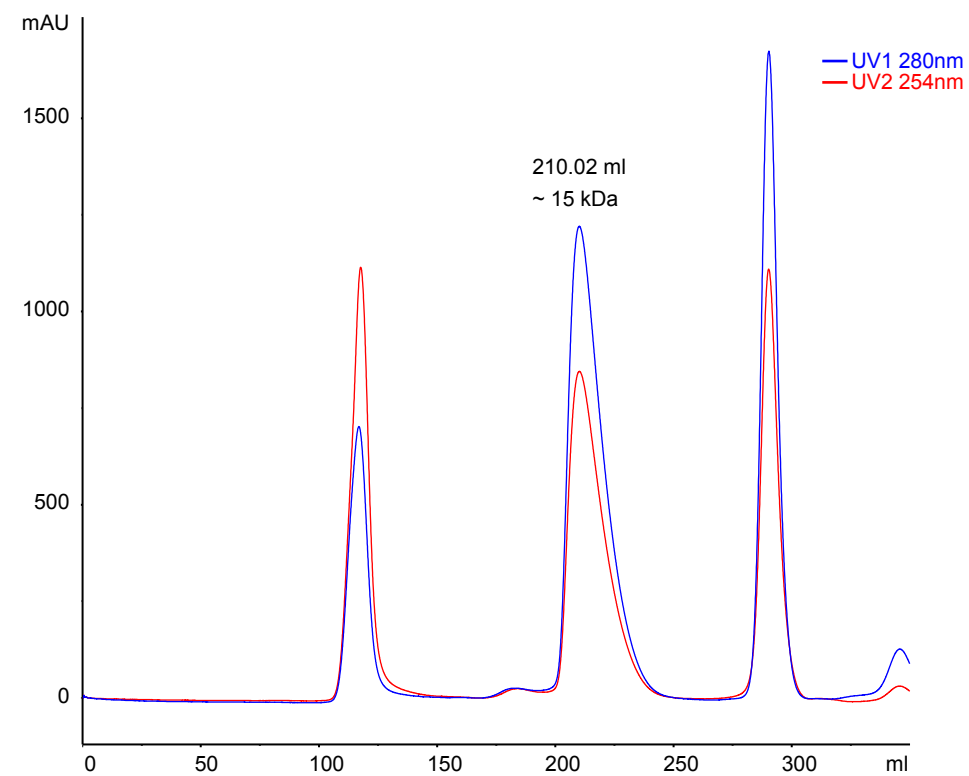

Supplement: Supplementary Data [file gky1011_supplemental_files.zip › Nishimura et al., 2018 Figure S1.pdf]

**A**

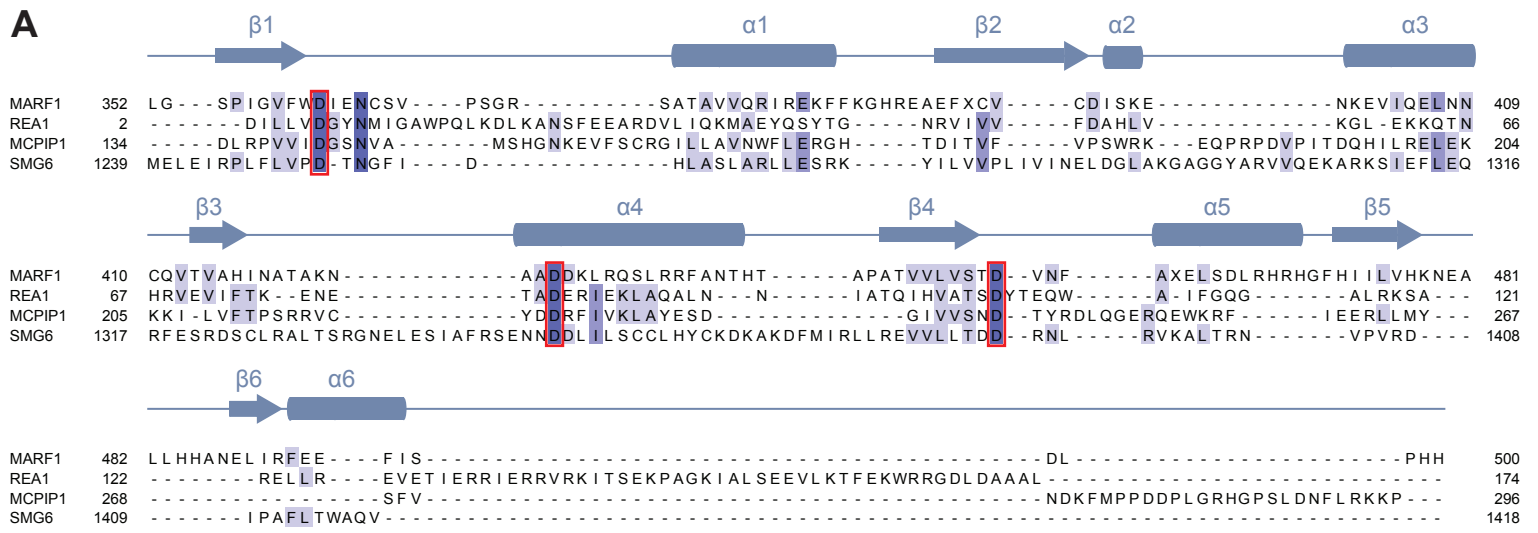

**B**

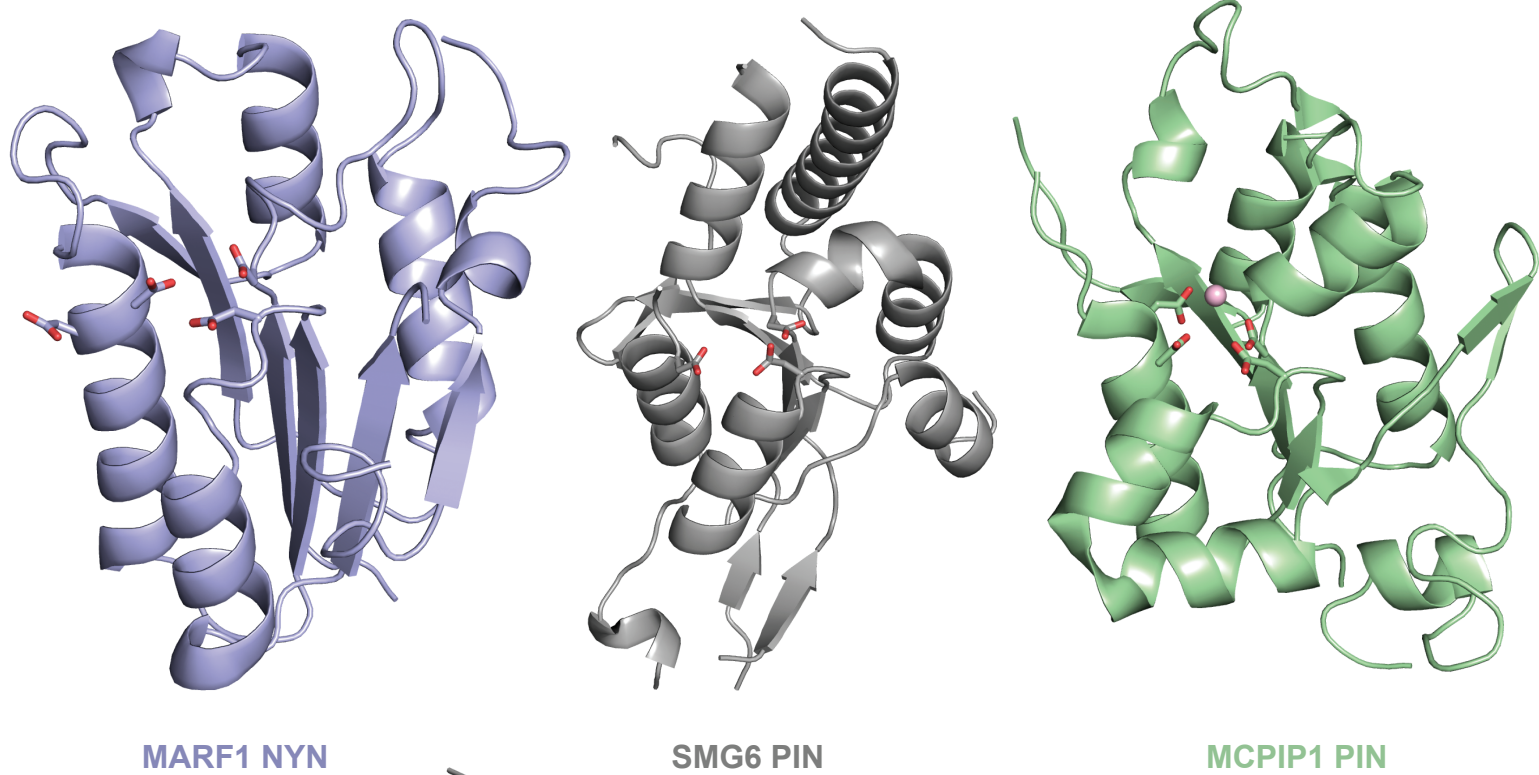

**C**

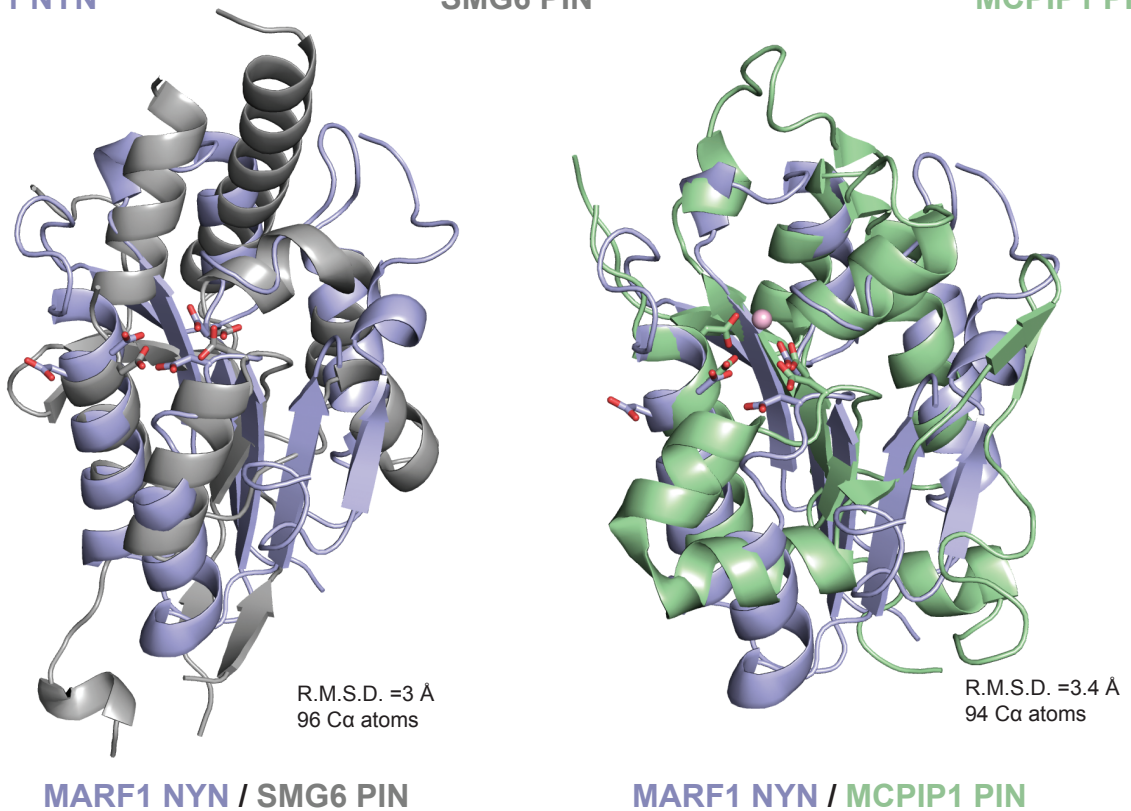

Supplement: Supplementary Data [file gky1011_supplemental_files.zip › Nishimura et al., 2018 Figure S2.pdf]
